# Supplementary material for: Large room temperature spin-to-charge conversion signals in a few-layer graphene/Pt lateral heterostructure
Source: Nat Commun. 2017 Sep 22;8:661. doi: 10.1038/s41467-017-00563-y (PMC5610249; doi:10.1038/s41467-017-00563-y)
Supplement: Supplementary file 1 — Supplementary Information [file 41467_2017_563_MOESM1_ESM.pdf]

Description of Supplementary Files

File Name: Supplementary Information
Description: Supplementary Notes, Supplementary Figures and Supplementary References

File Name: Peer Review File

Supplementary Note 1: Spin transport properties of graphene

The spin transport properties of graphene are obtained from the fitting of the Hanle measurement. The decoherence of the spin during precession causes the decay of an oscillating signal, which can be fitted using the following equation[1]:

$$R_{\mathrm{NL}} = -2R_{\mathrm{N}}\left(\frac{P_{\mathrm{F1}}}{1-P_{\mathrm{F1}}^2}\frac{R_{\mathrm{F1}}}{R_{\mathrm{N}}}+\frac{P_{\mathrm{I1}}}{1-P_{\mathrm{I1}}^2}\frac{R_{\mathrm{I1}}}{R_{\mathrm{N}}}\right)\left(\frac{P_{\mathrm{F2}}}{1-P_{\mathrm{F2}}^2}\frac{R_{\mathrm{F2}}}{R_{\mathrm{N}}}+\frac{P_{\mathrm{I2}}}{1-P_{\mathrm{I2}}^2}\frac{R_{\mathrm{I2}}}{R_{\mathrm{N}}}\right)\frac{C_{12}}{\det(\check{X})}\,, \tag{1}$$

where $R_{\mathrm{F}k} = \rho_{\mathrm{F}}\lambda_{\mathrm{F}}/A_{\mathrm{I}k}$ are the spin resistances of the kth FM contact (k = 1 is the injector and k = 2 is the detector), with resistivity ρ_{F}, spin diffusion length λ_{F} and contact area $A_{\mathrm{I}k}$; P_{Fk} are the spin polarizations of the kth FM contact; $R_{\mathrm{N}} = \frac{R_{\mathrm{Gr}}^{\blacksquare}\lambda_{\mathrm{Gr}}}{w_{\mathrm{Gr}}}$ is the spin resistance of graphene calculated with its sheet resistance ($R_{\mathrm{Gr}}^{\blacksquare}$), spin diffusion length (λ_{Gr}) and width (w_{Gr}); $R_{\mathrm{I}k} = 1/G_{\mathrm{I}k}$ is the resistance of the kth interface, where $G_{\mathrm{I}k} = G_{\mathrm{I}k}^{\uparrow} + G_{\mathrm{I}k}^{\downarrow}$ is the conductance of the kth interface that considers both spin up and down channels; $P_{\mathrm{I}k} = \left(G_{\mathrm{I}k}^{\uparrow} - G_{\mathrm{I}k}^{\downarrow}\right)/\left(G_{\mathrm{I}k}^{\uparrow} + G_{\mathrm{I}k}^{\downarrow}\right)$ describes the interfacial spin polarization; and C_{12} and $\det\left(\check{X}\right)$ are defined as[1]:

$$C_{12} = -\det\begin{pmatrix}\mathrm{Re}\left[\bar{\lambda}_{\omega}e^{-L/\tilde{\lambda}_{\omega}}\right] & -\mathrm{Im}\left[\bar{\lambda}_{\omega}e^{-L/\tilde{\lambda}_{\omega}}\right] & -\mathrm{Im}\left[\bar{\lambda}_{\omega}\right]\\ \mathrm{Im}\left[\bar{\lambda}_{\omega}\right] & r_{1\perp}+\mathrm{Re}\left[\bar{\lambda}_{\omega}\right] & \mathrm{Re}\left[\bar{\lambda}_{\omega}e^{-L/\tilde{\lambda}_{\omega}}\right]\\ \mathrm{Im}\left[\bar{\lambda}_{\omega}e^{-L/\tilde{\lambda}_{\omega}}\right] & \mathrm{Re}\left[\bar{\lambda}_{\omega}e^{-L/\tilde{\lambda}_{\omega}}\right] & r_{2\perp}+\mathrm{Re}\left[\bar{\lambda}_{\omega}\right]\end{pmatrix}, \tag{2}$$

$$\check{X} = \begin{pmatrix} r_{1\parallel}+\mathrm{Re}\left[\bar{\lambda}_{\omega}\right] & \mathrm{Re}\left[\bar{\lambda}_{\omega}e^{-L/\tilde{\lambda}_{\omega}}\right] & -\mathrm{Im}\left[\bar{\lambda}_{\omega}\right] & -\mathrm{Im}\left[\bar{\lambda}_{\omega}e^{-L/\tilde{\lambda}_{\omega}}\right]\\ \mathrm{Re}\left[\bar{\lambda}_{\omega}e^{-L/\tilde{\lambda}_{\omega}}\right] & r_{2\parallel}+\mathrm{Re}\left[\bar{\lambda}_{\omega}\right] & -\mathrm{Im}\left[\bar{\lambda}_{\omega}e^{-L/\tilde{\lambda}_{\omega}}\right] & -\mathrm{Im}\left[\bar{\lambda}_{\omega}\right]\\ \mathrm{Im}\left[\bar{\lambda}_{\omega}\right] & \mathrm{Im}\left[\bar{\lambda}_{\omega}e^{-L/\tilde{\lambda}_{\omega}}\right] & r_{1\perp}+\mathrm{Re}\left[\bar{\lambda}_{\omega}\right] & \mathrm{Re}\left[\bar{\lambda}_{\omega}e^{-L/\tilde{\lambda}_{\omega}}\right]\\ \mathrm{Im}\left[\bar{\lambda}_{\omega}e^{-L/\tilde{\lambda}_{\omega}}\right] & \mathrm{Im}\left[\bar{\lambda}_{\omega}\right] & \mathrm{Re}\left[\bar{\lambda}_{\omega}e^{-L/\tilde{\lambda}_{\omega}}\right] & r_{2\perp}+\mathrm{Re}\left[\bar{\lambda}_{\omega}\right]\end{pmatrix}, \tag{3}$$

where $\bar{\lambda}_{\omega} = \tilde{\lambda}_{\omega}/\lambda_{\mathrm{N}}$ with $\tilde{\lambda}_{\omega} = \frac{\lambda_{\mathrm{N}}}{\sqrt{i+i\omega_{\mathrm{L}}\tau_{\mathrm{sf}}}}$ and the Larmor frequency $\omega_{\mathrm{L}} = \gamma_e B_{\perp} = \frac{g\mu_{\mathrm{B}}}{\hbar}B_{\perp}$; $r_{k\parallel} = \left(\frac{2}{1-P_{\mathrm{I}k}^2}\frac{R_{\mathrm{I}k}}{R_{\mathrm{N}}}+\frac{2}{1-P_{\mathrm{F}k}^2}\frac{R_{\mathrm{F}k}}{R_{\mathrm{N}}}\right)$; L is the center-to-center distance between FM electrodes; and $r_{k\perp} = \frac{1}{R_{\mathrm{N}}G_{\mathrm{I}k}^{\uparrow\downarrow}}$ with $G_{\mathrm{I}k}^{\uparrow\downarrow}$ being the spin mixing interface conductance.

The Hanle measurement of the reference graphene LSV (Fig. 2c of the main text) also contains the effect of the rotation of the Co magnetizations with the external magnetic field (B_y), which tends to align the spin polarization with B_y, restoring the R_{NL} signal to its zero-field value $R_{\mathrm{NL}}(0)$ for parallel Co magnetizations. When this effect is taken into account, R_{NL} can be expressed as[2,3]:

$$R_{\mathrm{NL}}^{\mathrm{P(AP)}}\left(B_y,\theta\right) = \pm R_{\mathrm{NL}}^{\mathrm{P}}\left(B_y\right)\cos^2(\theta) + |R_{\mathrm{NL}}(0)|\sin^2(\theta)\,, \tag{4}$$

where $R_{\mathrm{NL}}^{\mathrm{P(AP)}}$ is the non-local resistance measured as a function of B_y when the two Co electrodes are parallel (P) or antiparallel (AP) and θ is the angle of the Co magnetization with respect to the easy axis of the electrode (x axis). Note that the sign "+" corresponds to the P curve and "–" to the AP curve, and that $R_{\mathrm{NL}}^{\mathrm{P}}\left(B_y\right) = -R_{\mathrm{NL}}^{\mathrm{AP}}\left(B_y\right)$ for the pure spin precession and

decoherence. By the proper combination of the measured P and AP curves, we can obtain the rotation of the Co magnetization (Fig. 2d of the main text):

$$\sin^2(\theta) = \frac{R_{\mathrm{NL}}^{\mathrm{P}}(B_y,\theta)+R_{\mathrm{NL}}^{\mathrm{AP}}(B_y,\theta)}{2|R_{\mathrm{NL}}(0)|}, \quad (5)$$

and the pure spin precession and decoherence (Fig.2e of the main text):

$$R_{\mathrm{NL}}^{\mathrm{P}}(B_y) = |R_{\mathrm{NL}}(0)| \frac{R_{\mathrm{NL}}^{\mathrm{P}}(B_y,\theta)-R_{\mathrm{NL}}^{\mathrm{AP}}(B_y,\theta)}{2|R_{\mathrm{NL}}(0)|-R_{\mathrm{NL}}^{\mathrm{P}}(B_y,\theta)-R_{\mathrm{NL}}^{\mathrm{AP}}(B_y,\theta)}. \quad (6)$$

For the fitting of the pure spin precession and decoherence curve of the reference graphene LSV in Fig. 2e, we assume the injecting and detecting electrodes have the same spin polarizations ($P_{\mathrm{Co1}} = P_{\mathrm{Co2}} = P_{\mathrm{Co}}$ and $P_{\mathrm{ICo1}} = P_{\mathrm{ICo2}} = P_{\mathrm{ICo}}$) and, following ref. 1, we assume an isotropic spin absorption, hence $G_{\mathrm{I}k}^{\uparrow\downarrow} = 1/(2R_{\mathrm{I}k} + 2R_{\mathrm{F}k})$. We fix the following experimental parameters: P_{Co} = 0.12 (ref. 4), R_{ICo1} = 42 kΩ, R_{ICo2} = 10 kΩ, L = 2.7 μm, w_{Gr} = 250 nm, w_{Co1} = 344 nm, w_{Co2} = 315 nm, $R_{\mathrm{Gr}}^{\blacksquare}$ = 1085 Ω, ρ_{Co} = 19 μΩcm (ref. 4), λ_{Co} = 40 nm (ref. 5,6), and obtain P_{ICo} = 0.068 ± 0.001, D = 0.005 m^2s^{-1}, and λ_{Gr} = 1.20 ± 0.02 μm. Because the spin signal is constant across the temperature range from 10 K to 300 K, we assume the spin diffusion length of graphene is independent of temperature.

Supplementary Note 2: Control experiments

In order to rule out any spurious magnetoresistance effect in graphene as the origin of the observed ISHE signal, we fabricated a control device where we substitute the Pt wire with a Cu wire, which has a weak spin-orbit coupling and, therefore, no spin-to-charge conversion signal is expected[7,8]. As the dimensions of the control device are very similar to those of the Pt/graphene device in the main manuscript, any spurious effect other than the ISHE signal, such as magnetoresistive effects arising from the stray fields created by the Co injector, should also be present in the control measurement. First of all, we check that the Co electrode is of similar quality as the Pt/graphene sample by measuring a reference spin valve in a nonlocal configuration. The nice and clear nonlocal spin signal indicates that the Co electrode next to the Cu wire is an efficient spin injector. Next, we measure the voltage drop across the Cu wire while using the Co electrode for spin injection in the ISHE measurement configuration. The result is shown in Supplementary Fig. 1b. This measurement produces a flat nonlocal background much smaller than that of the ISHE signal measured in the device presented in the main text (Device #1), indicating there is no spurious contribution to the ISHE signal (compare black and blue curves in Supplementary Fig. 1b). The same control experiments were carried out in a total of five control devices and all of them showed a similar flat background.

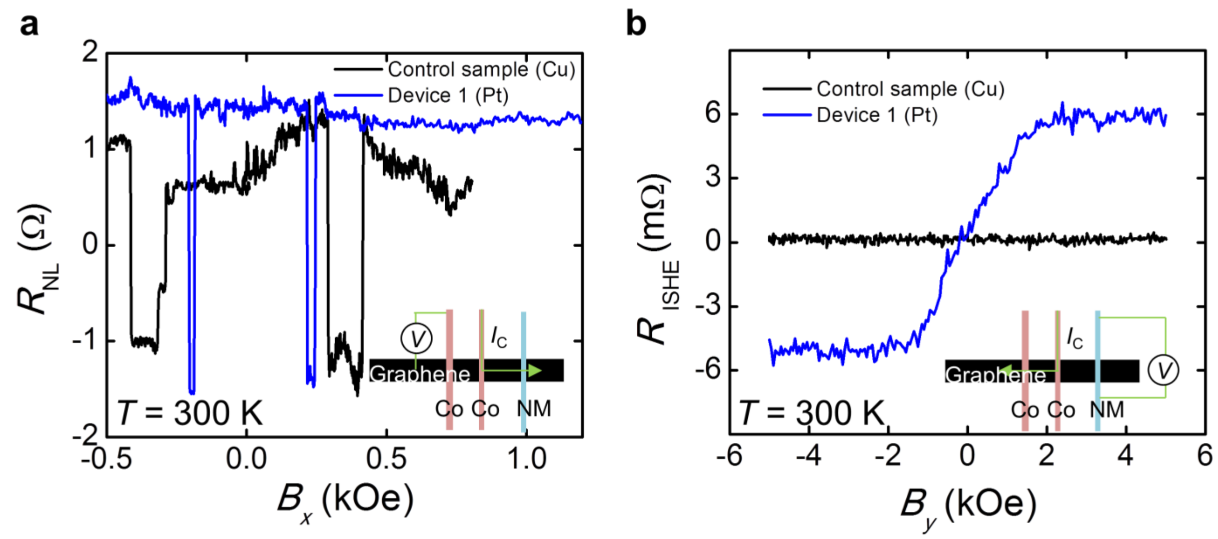

Supplementary Figure 1 | Comparison between Device #1 and a control device using graphene lateral spin valves and Cu wire. (a) Nonlocal magnetoresistance as a function of B_x using a reference spin valve next to the non-magnetic metal wire shows similar spin signal in both the control sample and Device #1, indicating that Co electrode next to Cu wire in the control device is a spin injector as good as in Device #1. I_C = 10 μA and T = 300 K in both measurements. (b) ISHE resistance as a function of B_y measured in both the control device and Device #1. I_C = 10 μA and T = 300 K in both measurements.

In order to rule out further spurious effects such as drift due to heating, we performed trace and retrace of the ISHE measurements by increasing and decreasing the applied magnetic field for each temperature. An example of this measurement from Device #1 at 300 K is shown in Supplementary Fig. 2. The overlapping of the trace and retrace curves rules out any drift due to heating.

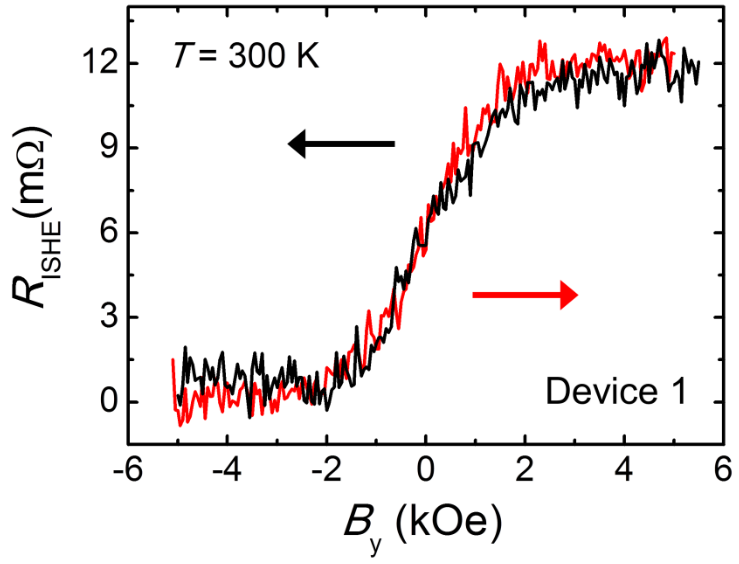

Supplementary Figure 2 | ISHE resistance for increasing and decreasing magnetic field. ISHE resistance as a function of B_y with I_C = 10 μA and T = 300 K measured in Device #1. Red solid curve corresponds to the increasing field sweep and black solid curve to the decreasing field sweep. Note that the figure plots the raw data without any baseline subtraction.

Supplementary Note 3: Reproducibility

The key results we presented in this manuscript, which is the SHE and ISHE effect in Pt and its temperature dependence, are fully reproducible among different samples. Here, we show a second device (#2), in which we measure the SHE and ISHE effects at 300 K by simply swapping the current and voltage probes, demonstrating that they are reciprocal to each other (see Supplementary Fig. 3a). The ΔR_{SCC} signal increases with temperature, reaching the maximum value at 300 K (Supplementary Fig. 3b).

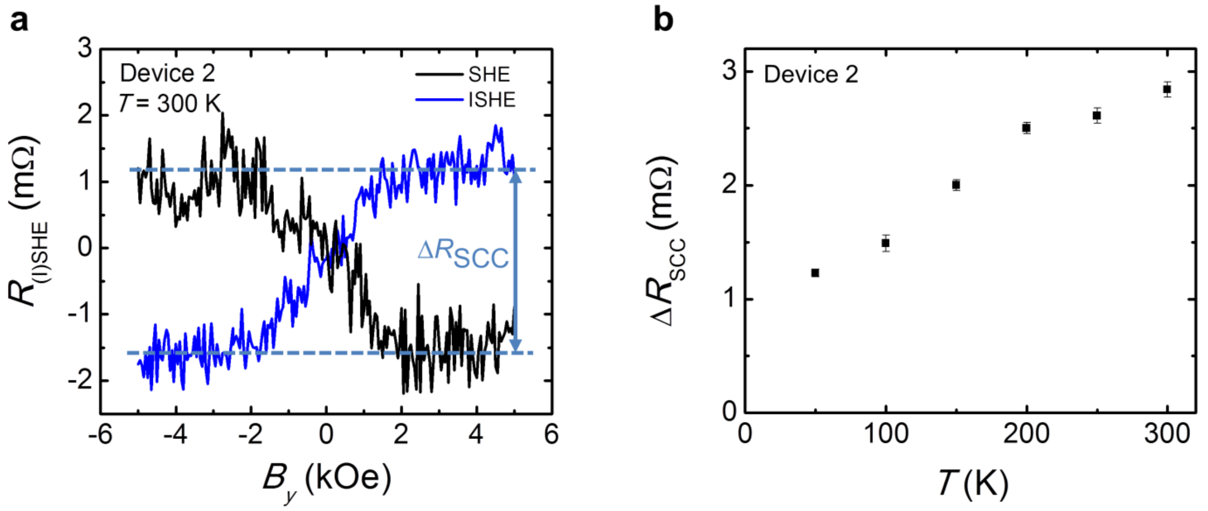

Supplementary Figure 3 | Spin-to-charge conversion signal in another graphene/Pt lateral heterostructure. (a) SHE (black) and ISHE (blue) resistance as a function of B_y measured with I_{C} = 10 μA at 300 K in Device #2, demonstrating the reciprocity of these two effects. A baseline signal of 19 mΩ (18 mΩ) has been subtracted from the ISHE (SHE) curve. (b) Temperature dependence of the ΔR_{SCC} signal in Device #2. Error bars are calculated using the standard errors associated with the statistical average of the nonlocal resistance at positive and negative saturated magnetic fields.

The magnitude of the ΔR_{SCC} signal measured in Device #2 is smaller than the device presented in the main text (#1) (compare Supplementary Fig. 3b with Fig. 4d), although the dimensions are very similar (the widths of the Co injector and the Pt wire in Device #2 are 336 nm and 193 nm, respectively). This is due to the variation of the interface resistance between Co and graphene, i.e., R_{ICo1} in Device #2 is smaller (2 kΩ) than in Device #1 (14.7 kΩ). Despite this variation, both the ΔR_{SCC} signal and its temperature dependence can be fully explained with the spin diffusion model (Supplementary Equations 8 and 9), evidencing the robustness of the performance of the graphene/Pt lateral heterostructures.

Supplementary Note 4: Spin absorption by Pt

The Pt wire placed in between the two Co electrodes has a much smaller spin resistance than the graphene channel. Therefore, the presence of Pt will cause an additional relaxation to the spins transported in the graphene channel. This additional spin relaxation, which shows up as a decrease of measured spin signal, is called spin absorption. According to the standard one-dimensional (1D) spin diffusion model[9], the measured spin signal after spin absorption can be calculated using the following equation:

$$\Delta R_{\mathrm{NL}}^{\mathrm{abs}} = 4R_{\mathrm{N}}\hat{\alpha}_1\hat{\alpha}_2 \frac{(r_3-1)e^{-\frac{L}{\lambda_{\mathrm{N}}}}}{r_1r_2(r_3-Q_{\mathrm{I3}})-r_1(1+Q_{\mathrm{I3}})e^{-\frac{2(L-d)}{\lambda_{\mathrm{N}}}}-r_2(1-Q_{\mathrm{I3}})e^{-\frac{2d}{\lambda_{\mathrm{N}}}}-(r_3-Q_{\mathrm{I3}})e^{-\frac{2L}{\lambda_{\mathrm{N}}}}+2e^{-\frac{2L}{\lambda_{\mathrm{N}}}}}, \quad (7)$$

with $r_k = 2Q_{\mathrm{I}k} + 2Q_{\mathrm{F}k} + 1$, $\hat{\alpha}_k = P_{\mathrm{I}k}Q_{\mathrm{I}k} + P_{\mathrm{F}k}Q_{\mathrm{F}k}$, $Q_{\mathrm{I}k} = \frac{1}{1-P_{\mathrm{I}k}^2}\frac{R_{\mathrm{I}k}}{R_{\mathrm{N}}}$, and $Q_{\mathrm{F}k} = \frac{1}{1-P_{\mathrm{F}k}^2}\frac{R_{\mathrm{F}k}}{R_{\mathrm{N}}}$, where $k = 1,2,3$ refers to the FM injector (F1), the FM detector (F2) and middle metallic wire (M), respectively. d is the distance between the FM injector and the middle wire. The spin resistance of the middle wire is defined as $R_{\mathrm{F3}} = R_{\mathrm{M}} = \frac{\rho_{\mathrm{M}}\lambda_{\mathrm{M}}}{w_{\mathrm{M}}w_{\mathrm{N}}\, tanh[t_{\mathrm{M}}/\lambda_{\mathrm{M}}]}$. The geometrical factors w_{M} and t_{M} are the width and thickness of the middle wire, being λ_{M} its spin diffusion length. When w_{M} becomes comparable to λ_{N}, Supplementary Equation 7 is not accurate anymore, as discussed in ref. 10). However, this is not the case in our samples, because the spin diffusion length of our graphene is much longer (λ_{Gr} = 1.20 ± 0.02 μm) than the width of the Pt wire (w_{Pt} = 198 nm) and, thus, the use of the 1D model is valid. Experimentally, this validity has already been proven in devices with Pt spin absorber and Cu spin transport channel[11]. Table 1 in ref. 11 shows that the obtained spin diffusion length and spin Hall angle using the 1D model have a very small deviation with respect to the 3D model, confirming the validity of our approach.

We assume the FM injector and detector spin polarizations are identical ($P_{\mathrm{Co1}} = P_{\mathrm{Co2}} = P_{\mathrm{Co}}$ and $P_{\mathrm{ICo1}} = P_{\mathrm{ICo2}} = P_{\mathrm{ICo}}$), but we consider their different interface resistances. Therefore, we can write for our case:

$r_1 = 2Q_{\mathrm{ICo1}} + 2Q_{\mathrm{Co1}} + 1,$
$r_2 = 2Q_{\mathrm{ICo2}} + 2Q_{\mathrm{Co2}} + 1,$
$r_3 = 2Q_{\mathrm{IPt}} + 2Q_{\mathrm{Pt}} + 1,$
$\hat{\alpha}_1 = P_{\mathrm{ICo}}Q_{\mathrm{ICo1}} + P_{\mathrm{Co}}Q_{\mathrm{Co1}},$
$\hat{\alpha}_2 = P_{\mathrm{ICo}}Q_{\mathrm{ICo2}} + P_{\mathrm{Co}}Q_{\mathrm{Co2}}$

where the subscripts ICo1 and ICo2 stand for the Co injector/graphene and Co detector/graphene interface, respectively, and the subscript IPt for the Pt/graphene interface.

Taking into account that $Q_{\mathrm{Co1}}, Q_{\mathrm{Co2}} \ll Q_{\mathrm{ICo1}}, Q_{\mathrm{ICo2}}$, that $Q_{\mathrm{IPt}}, Q_{\mathrm{Pt}} \ll 1$, and considering that $L = 2d$, Supplementary Equation 7 can be simplified to:

$$\Delta R_{\mathrm{NL}}^{\mathrm{abs}} = 8R_{\mathrm{Gr}}Q_{\mathrm{ICo1}}Q_{\mathrm{ICo2}}P_{\mathrm{ICo}}^2 \frac{(Q_{\mathrm{IPt}}+Q_{\mathrm{Pt}})e^{-\frac{L}{\lambda_{\mathrm{Gr}}}}}{(2Q_{\mathrm{ICo1}}+1)(2Q_{\mathrm{ICo2}}+1)-2(Q_{\mathrm{ICo1}}+Q_{\mathrm{ICo2}}+1)e^{-\frac{L}{\lambda_{\mathrm{Gr}}}}+e^{-\frac{2L}{\lambda_{\mathrm{Gr}}}}}. \quad (8)$$

We fix the following parameters in the above equation: P_{ICo} = 0.068, λ_{Gr} = 1.20 μm, $R_{\mathrm{Gr}}^{\blacksquare}$ = 1085 Ω, L = 1.27 μm, w_{Gr} = 250 nm, w_{Pt} = 198 nm, R_{ICo1} = 14.7 kΩ, R_{ICo2} = 15 kΩ, ρ_{Pt} = 99 μΩ·cm (50 K) and 134 μΩ·cm (300 K) and t_{Pt} = 21 nm. We are left with two parameters that are crucial for the spin absorption: λ_{Pt} and R_{IPt}.

We measured directly the interface resistance between Pt and graphene, R_{IPt} by using a 4-point configuration in the graphene/Pt cross-shaped junction. The measured values are negative, ranging from -8.5 Ω (10 K) to -13 Ω (300 K). This is an artifact which occurs when the resistance of the channel is of the order or higher than the interface resistance due to an inhomogeneous current distribution in this geometry, which is expected due to the large sheet

resistance of graphene[12,13]. Its precise value can be determined when combining the results of the spin absorption described by Supplementary Equation 8 with the results of the ISHE experiments described by Supplementary Equation 9 (see Supplementary Note 5).

Supplementary Note 5: Inverse spin Hall effect by Pt

The spin-to-charge conversion signal ΔR_{SCC} of the ISHE experiment is given by the following expression [7,14,15]:

$$\Delta R_{\mathrm{SCC}} = \frac{2\theta_{\mathrm{SH}}\rho_{\mathrm{M}}x_{\mathrm{M/N}}}{w_{\mathrm{M}}}\left(\frac{\bar{I_{\mathrm{S}}}}{I_{\mathrm{C}}}\right), \quad (9)$$

where θ_{SH} is the spin Hall angle of the middle wire (M) and $x_{\mathrm{M/N}}$ is the correction factor that considers the current in M shunted through the non-magnetic channel (N) (ref. 7). $\bar{I_{\mathrm{S}}}$ is the effective spin current injected vertically into the M wire that contributes to the ISHE, because the spin current at the M/N interface $I_{\mathrm{S}}(z=0)$ is diluted into the M thickness. To calculate $\bar{I_{\mathrm{S}}}$, we integrate the spin current injected into the M wire and then divide it by the M thickness [7, 14,15]:

$$\frac{\bar{I_{\mathrm{S}}}}{I_{\mathrm{C}}} \equiv \frac{\int_0^{t_{\mathrm{M}}} I_{\mathrm{S}}(z)dz}{t_{\mathrm{M}}I_{\mathrm{C}}} = \frac{\lambda_{\mathrm{M}}}{t_{\mathrm{M}}}\frac{\left(1-e^{-\frac{t_{\mathrm{M}}}{\lambda_{\mathrm{M}}}}\right)^2}{1-e^{-\frac{2t_{\mathrm{M}}}{\lambda_{\mathrm{M}}}}}\frac{I_{\mathrm{S}}(z=0)}{I_{\mathrm{C}}}, \quad (10)$$

where $I_{\mathrm{S}}(z=0)$ can be calculated using the following equation:

$$\frac{I_{\mathrm{S}}(z=0)}{I_{\mathrm{C}}} = \frac{2\hat{\alpha}_1\left[r_2(1-Q_{\mathrm{I3}})e^{-\frac{d}{\lambda_{\mathrm{N}}}}-(1+Q_{\mathrm{I3}})e^{-\frac{(2L-d)}{\lambda_{\mathrm{N}}}}\right]}{r_1r_2(r_3-Q_{\mathrm{I3}})-r_1(1+Q_{\mathrm{I3}})e^{-\frac{2(L-d)}{\lambda_{\mathrm{N}}}}-r_2(1-Q_{\mathrm{I3}})e^{-\frac{2d}{\lambda_{\mathrm{N}}}}-(r_3-Q_{\mathrm{I3}})e^{-\frac{2L}{\lambda_{\mathrm{N}}}}+2e^{-\frac{2L}{\lambda_{\mathrm{N}}}}}. \quad (11)$$

Taking into account that $Q_{\mathrm{Co1}}, Q_{\mathrm{Co2}} \ll Q_{\mathrm{ICo1}}, Q_{\mathrm{ICo2}}$, that $Q_{\mathrm{IPt}}, Q_{\mathrm{Pt}} \ll 1$, and considering that $L = 2d$, Supplementary Equation 10 can be simplified to:

$$\frac{\bar{I_{\mathrm{S}}}}{I_{\mathrm{C}}} = \frac{\lambda_{\mathrm{Pt}}}{t_{\mathrm{Pt}}}\frac{\left(1-e^{-\frac{t_{\mathrm{Pt}}}{\lambda_{\mathrm{Pt}}}}\right)^2}{1-e^{-\frac{2t_{\mathrm{Pt}}}{\lambda_{\mathrm{Pt}}}}}\frac{2P_{\mathrm{ICo}}Q_{\mathrm{ICo1}}\left[(2Q_{\mathrm{ICo2}}+1)e^{\frac{L}{2\lambda_{\mathrm{Gr}}}}-e^{-\frac{L}{2\lambda_{\mathrm{Gr}}}}\right]}{(2Q_{\mathrm{ICo1}}+1)(2Q_{\mathrm{ICo2}}+1)e^{\frac{L}{\lambda_{\mathrm{Gr}}}}-2(Q_{\mathrm{ICo1}}+Q_{\mathrm{ICo2}}+1)+e^{-\frac{L}{\lambda_{\mathrm{Gr}}}}}. \quad (12)$$

In the case of Pt/graphene cross junction, the equivalent sheet resistance of graphene and Pt is 1085 Ω and 64 Ω at 300 K, respectively. Therefore, the shunting coefficient $x_{\mathrm{Pt/Gr}}$ is expected to be very close to unity. Using $x_{\mathrm{Pt/Gr}}$ = 1, θ_{SH} = 17.8 ± 2.0% (50 K) and 23.4 ± 2.5% (300 K) from ref. 14, and the parameters used for Supplementary Equation S8 (Supplementary Note 4), we are left with the same two parameters: λ_{Pt} and R_{IPt}.

By using the experimental results from the spin absorption [$\Delta R_{\mathrm{NL}}^{\mathrm{abs}}$ = 24.5 ± 0.9 mΩ (50 K) and 22 ± 1 mΩ (300 K)] and ISHE experiments [ΔR_{SCC} = 5.9 ± 0.2 mΩ (50 K) and 11.2 ± 0.7 mΩ (300 K)], we can solve Supplementary Equations 8 and 9 simultaneously to extract the unknown values for λ_{Pt} and R_{IPt}. We obtain λ_{Pt} = 2.1 ± 0.3 nm and R_{IPt} = 10.6 ± 0.4 Ω at 50 K and λ_{Pt} = 2.1 ± 0.4 nm and R_{IPt} = 8.4 ± 0.4 Ω at 300 K.

Supplementary Note 6: Efficiency of spin injection and detection using Pt wires: Experiments and discussion

We prepared several samples (see, for instance, Supplementary Fig. 4a) with adjacent Pt electrodes to observe generation and detection of spin currents using SHE and ISHE. Unfortunately, a very small signal (~0.01 mΩ) is expected, due to the conductivity mismatch of the two Pt/graphene contacts (instead of one contact only in the cases of spin detection with ISHE or spin injection with SHE reported in the main text). In order to observe a spin signal from the Ohmic baseline in the non-local measurement, a magnetic field of 7 kOe is rotated in plane. In the *x*-direction, the dephasing of the Hanle precession would cancel the spin signal, while in the *y*-direction (the same as the spin polarization), no Hanle effect would occur. A $\cos^2$ dependence would be expected, with an amplitude corresponding to the spin signal. The noise of the measurement (0.1-0.2 mΩ) is larger than the expected signal and, therefore, cannot be observed (Supplementary Fig. 4b).

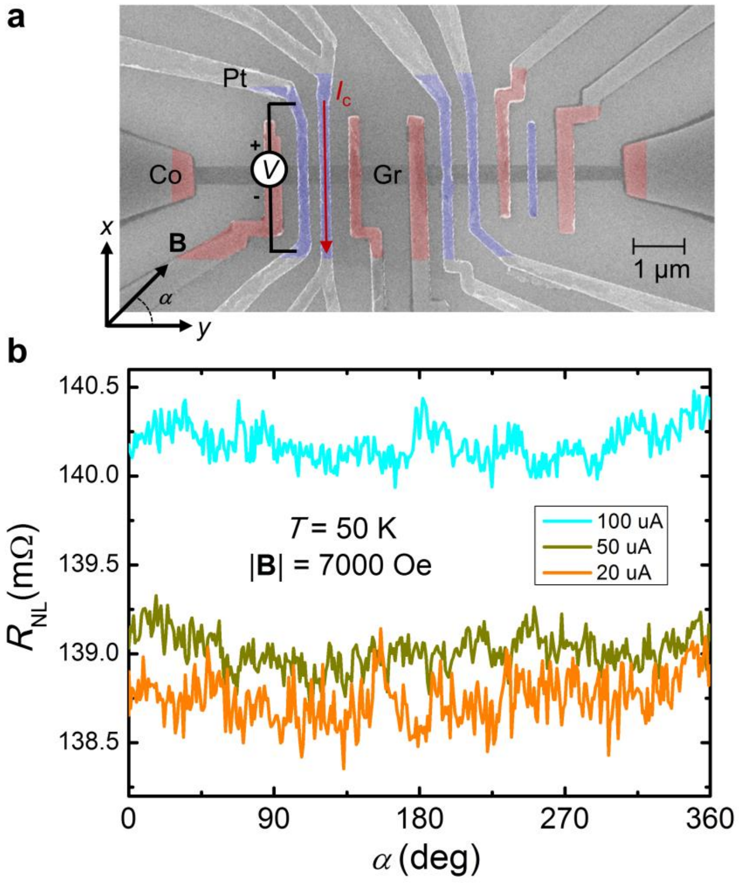

Supplementary Figure 4 | Simultaneous spin injection and detection in a graphene channel using Pt wires. (a) SEM image of a graphene/Pt lateral heterostructure with adjacent Pt electrodes (blue color) in a trilayer graphene channel (grey color). Ferromagnetic Co electrodes with TiO_2 barrier (red color) placed adjacent to each Pt are used to confirm proper spin injection via SHE or detection via ISHE of the Pt wires following the measurements described in the main text. The measurement configuration shown allows full spin injection and detection using the Pt wires. (b) Non-local resistance as a function of the angle of the applied magnetic field, measured using the configuration shown in (a) at *T*=50 K and **|B|**=7 kOe with different applied currents.

These results show that a full spin injection and detection with Pt is not useful at this stage due to the low efficiency for spin injection. Nevertheless, in our proof-of-principle device, we showed not only that there is spin injection to graphene using Pt, but also that the overall spin-to-charge conversion of the whole device is more efficient than in conventional lateral spin valves with metallic spin channel. One should be careful when directly comparing the non-local ΔR_{NL} signal and the spin-to-charge conversion ΔR_{SCC} signal. The former only probes the spin accumulation in the channel (in this case graphene) through a tunnel barrier or high resistive interface leading to a large voltage drop, but it cannot be further utilized, for instance to convert it to charge current for cascading in a spin-based logic circuit or to directly switch a magnetic element via spin transfer torque of the pure spin current. This limitation is equivalent to that observed in the local magnetoresistance of a spin valve: a high resistive interface helps in the spin injection, but is detrimental for the spin detection, because the current cannot flow into the detector [see Fig. 3 in ref. 16]. On the other hand, the configuration of the spin-to-charge conversion consists of a transparent interface through which spins can be absorbed or injected. Here the transport is diffusive and the impedance mismatch plays a role. But the transparent interface is necessary to allow for absorption of the pure spin current in the spin Hall material, which is then converted to a charge current which can be potentially utilized. ΔR_{SCC} directly probes the charge current generated in the spin Hall metal.

On the application side, the combination of spin injection from one ferromagnetic element where the non-volatile information is stored and subsequent spin-to-charge current conversion in a non-magnetic element is important for cascading in potential applications such as the spin-orbit logic proposed by Intel[17]. Additionally, substituting a FM element by a NM electrode overcomes the necessity of controlling the relative magnetic orientation of a second ferromagnet when used as a detector. For instance, another potential application of our results would be in the spin-based magnetologic device proposed by H. Dery *et al.*, where a graphene spin channel is connected with 5 ferromagnetic electrodes for input, operation and reading out[18,19]. If some of the ferromagnetic electrodes in the magnetologic device can be substituted by a spin Hall metal, this will lead to the control of spin currents by charge current instead of the magnetization of the ferromagnet, as well as to cascading output voltages from one logic element to the next.

Supplementary References

1. Idzuchi, H., Fert, A. & Otani, Y. Revisiting the measurement of the spin relaxation time in graphene-based devices. *Phys. Rev. B* **91**, 241407(R) (2015).
2. Valenzuela, S. O. & Tinkham, M. Direct electronic measurement of the spin Hall effect. *Nature* **442**, 176-179 (2006).
3. Mihajlovic, G., Pearson, J. E., Bader, S. D. & Hoffmann A. Surface Spin Flip Probability of Mesoscopic Ag Wires. *Phys. Rev. Lett.* **104**, 237202 (2010).
4. Villamor, E., Isasa, M., Hueso, L. E. & Casanova, F. Temperature dependence of spin polarization in ferromagnetic metals using lateral spin valves. *Phys. Rev. B* **88**, 184411 (2013).
5. Piraux, L., Dubois, S., Fert, A. & Belliard, L. The temperature dependence of the perpendicular giant magnetoresistance in Co/Cu multilayered nanowires. *Eur. Phys. J. B* **4**, 413-420 (1998).
6. Reilly, A. C. *et al*. Giant magnetoresistance of current-perpendicular exchange-biased spin-valves of Co/Cu. *IEEE Trans. Magn.* **34**, 939-941 (1998).

7. Niimi, Y. *et al.* Extrinsic Spin Hall Effect Induced by Iridium Impurities in Copper. *Phys. Rev. Lett.* **106**, 126601 (2011).
8. Niimi, Y. *et al*. Giant Spin Hall Effect Induced by Skew Scattering from Bismuth Impurities inside Thin Film CuBi Alloys. *Phys. Rev. Lett*. **109**, 156602 (2012).
9. Takahashi, S. & Maekawa, S. Spin injection and detection in magnetic nanostructures. *Phys. Rev. B* **67**, 052409 (2003).
10. Laczkowski, P. *et al.* Evaluation of spin diffusion length of AuW alloys using spin absorption experiments in the limit of large spin-orbit interactions. *Phys. Rev. B* **92**, 214405 (2015).
11. Niimi, Y. *et al.* Extrinsic spin Hall effects measured with lateral spin valve structures. *Phys. Rev. B* **89**, 054401 (2014).
12. Pedersen, R. J. & Vernon, F. L. Effect of film resistance on low-impedance tunneling measurements. *Appl. Phys. Lett.* **10**, 29-31 (1967).
13. Pomeroy, J. M. & Grube, H. “Negative resistance” errors in four-point measurements of tunnel junctions and other crossed-wire devices. *J. Appl. Phys.* **105**, 094503 (2009).
14. Sagasta, E. *et al*. Tuning the spin Hall effect of Pt from the moderately dirty to the superclean regime. *Phys. Rev. B* **94**, 060412(R) **(**2016).
15. Morota, M. *et al*. Indication of intrinsic spin Hall effect in 4d and 5d transition metals. *Phys. Rev. B* **83**, 174405 (2011).
16. Fert, A. & Jaffrès, H. Conditions for efficient spin injection from a ferromagnetic metal into a semiconductor. *Phys. Rev. B* **64**, 184420 (2001).
17. Manipatruni, S., Nikonov, D. E. & Young, I. A. Spin-orbit logic with magnetoelectric nodes: a scalable charge mediated nonvolatile spintronic logic. Preprint at https://arxiv.org/abs/1512.05428 (2017).
18. Dery, H. *et al.* Spin-based logic in semiconductors for reconfigurable large-scale circuits. *Nature* **447**, 573-576 (2007).
19. Dery, H. *et al.* Nanospintronics based on magnetologic gates. *IEEE Trans. Electron Devices* **59**, 259-262 (2012).
